# A streamlined integrated system integrating lysate release, freeze-dried reagents for multiplex polymerase chain reaction, and intelligent analysis for TORCHes pathogen identification

**DOI:** 10.3389/fmicb.2026.1788209

**Published:** 2026-03-24

**Authors:** Miao Fu, Siying He, Yingchao Wu, Sisi Mao, Wei Chen, Na Zhang, Xin Chen, Jinhua Wang, Lingzhi Zhou, Xiang Xi, Junqi Wu, Huabin Wang, Yongjun Ma

**Affiliations:** 1 Department of Clinical Laboratory, Affiliated Jinhua Hospital, Zhejiang University School of Medicine, Jinhua, Zhejiang, China; 2 Shanghai Pudong New Area People’s Hospital, Shanghai, China; 3 Lanxi Traditional Chinese Medicine Hospital, Lanxi, Zhejiang, China; 4 School of Laboratory Medicine and Life Sciences, Wenzhou Medical University, Wenzhou, Zhejiang, China

**Keywords:** analysis of melting curves of multi-color probes, freeze-dried microspheres, intelligent interpretation, lysate, TORCHes pathogen

## Abstract

**Introduction:**

Current *Toxoplasma gondii* (TOX), Epstein–Barr virus (EBV), rubella virus, human cytomegalovirus (HCMV), and herpes simplex virus types I and II (HSV I and II) (TORCHes) diagnostic methods are limited by challenges, such as multi-step workflows and cold-chain dependency. This study aimed to develop an integrated “sample-in-result-out” system combining rapid nucleic acid release, room-temperature-stable multiplex polymerase chain reaction (PCR), and automated interpretation to improve diagnostic accuracy.

**Methods:**

We utilized a self-prepared lysis buffer for fast nucleic acid extraction from diverse sample types. Primers, probes, and reaction components for six TORCHes pathogens were fabricated into freeze-dried microspheres via vacuum freeze-drying, enabling single-tube multicolor melting curve analysis (MMCA). The companion software, TORCHes-MCAv1.0, was used to automate pathogen typing and result reporting. Evaluation metrics encompassed sensitivity, specificity, precision, stability, and anti-interference ability. The system was validated using 210 clinical samples compared to commercial single-target qPCR, with Sanger sequencing resolving any discrepancies.

**Results:**

Nucleic acid extraction and release were completed within 1 min. Freeze-dried microspheres demonstrated exceptional repeatability, with *T*_m_ intra- and inter-batch CVs of 0.03–0.18%. Limits of detection (LoD) were 200 copies/mL for HSV-I/II, HCMV, and TOX, and 500 copies/mL for EBV and RV. Specificity analysis showed no cross-reactivity with non-target pathogens. Stability testing confirmed the microspheres could be stored stably at room temperature for 1 year. Clinical validation showed near-perfect agreement with qPCR (Kappa = 0.965–1.000), with all seven discrepant results confirmed correct via sequencing. The TORCHes-MCAv1.0 software achieved 100% concordance with manual interpretation.

**Conclusion:**

We developed a robust, streamlined integrated TORCHes detection system. Its simplicity, stability, and high-throughput nature make it a valuable complementary tool for confirming active infection and differentiating pathogen subtypes in complex clinical scenarios.

## Introduction

TORCH is an umbrella term for a group of perinatal pathogens—*Toxoplasma gondii* (TOX), Rubella virus (RV), Human Cytomegalovirus (HCMV), Herpes Simplex Virus types I and II (HSV-I/II), and Epstein–Barr virus (EBV)—known for their ability to traverse the placental barrier (Neu et al., 2015; Melvin et al., 2022; Hutton et al., 2014). The term “TORCHes” is used herein to specifically denote this expanded panel, which includes EBV in addition to the classic TORCH pathogens (Toxoplasma, Rubella, Cytomegalovirus, and Herpes simplex). Although EBV is not part of the original TORCH acronym, it is explicitly included under the “others (O)” category in the widely adopted *Chinese Expert Consensus on TORCH detection*, acknowledging its potential for adverse pregnancy outcomes, such as miscarriage (Zhu et al., 2020). Supporting its relevance, studies have demonstrated a significant association between EBV infection and miscarriage in later pregnancy, and EBV DNA has been detected in neonatal cord blood, confirming the occurrence of maternal-fetal transmission (Mitsoda et al., 2014; Sloan et al., 2022). As a member of the TORCHes pathogen group, EBV shares the broader clinical risks characteristic of these infections: maternal infection, often asymptomatic or presenting with nonspecific symptoms, can lead to devastating consequences for the fetus or newborn, including spontaneous abortion, stillbirth, premature delivery, and a spectrum of congenital anomalies and neuro developmental disorders. Given the limited therapeutic options after infection, accurate and timely pathogen detection is paramount for risk assessment and clinical management (Paquet and Yudin, 2018; Saldan et al., 2017; Kilby et al., 2019).

Serological testing for pathogen-specific IgM and IgG antibodies serves as the cornerstone for TORCHes screening in pregnant women, as affirmed by professional guidelines including the Chinese Expert Consensus (Zhu et al., 2020). Nevertheless, interpreting serological results presents considerable complexities, largely stemming from the unavoidable window period before antibody seroconversion occurs, frequent cross-reactivity contributing to false-positive IgM readings, and the challenge in reliably differentiating active from past infections based on static antibody profiles alone (Wandinger et al., 2011; Chen et al., 2019). Against this backdrop, nucleic acid amplification tests (NAATs) play an indispensable complementary role. As emphasized in the Expert Consensus, NAATs provide critical diagnostic value in specific situations: verifying active infection in cases of inconclusive serology, identifying the causative pathogen in symptomatic neonates, and especially in differentiating between HSV-I and HSV-II, a distinction with substantial implications for clinical counselling and therapeutic decision-making. Quantitative real-time PCR (qPCR) offers high analytical sensitivity and specificity. However, conventional single conventional single-target qPCR faces notable constraints in comprehensively detecting all six TORCHes pathogens, primarily due to limited fluorescence channels requiring multiple separate reactions. Additionally, the strict dependence of liquid reagents on uninterrupted cold-chain logistics poses a fundamental barrier to wider dissemination, particularly beyond centralized laboratory environments (Xiang et al., 2020; Kang et al., 2004; Vinjé, 2015).

To bridge this technological gap, the integration of lyophilized reagent technology with multicolour melting curve analysis (MMCA) emerges as a promising strategy (Abla and Mehanna, 2022). Lyophilization enhances reagent stability, allowing for ambient temperature storage. MMCA, utilizing differentially labelled probes and *T*_m_ analysis, holds the potential for simultaneous detection and differentiation of multiple pathogens within a single closed tube. This significantly streamlines the workflow and reduces contamination risk (Fu et al., 2012; Li et al., 2022). Nonetheless, despite these clear benefits, the application of this combined methodology to comprehensive TORCHes panel detection remains relatively unexplored. A persistent obstacle lies in the interpretation of intricate multi-target melting curves, which currently depends heavily on manual assessment. Such subjectivity introduces the possibility of inconsistency and misjudgment, highlighting an area in urgent need of standardization.

**Figure 1:**
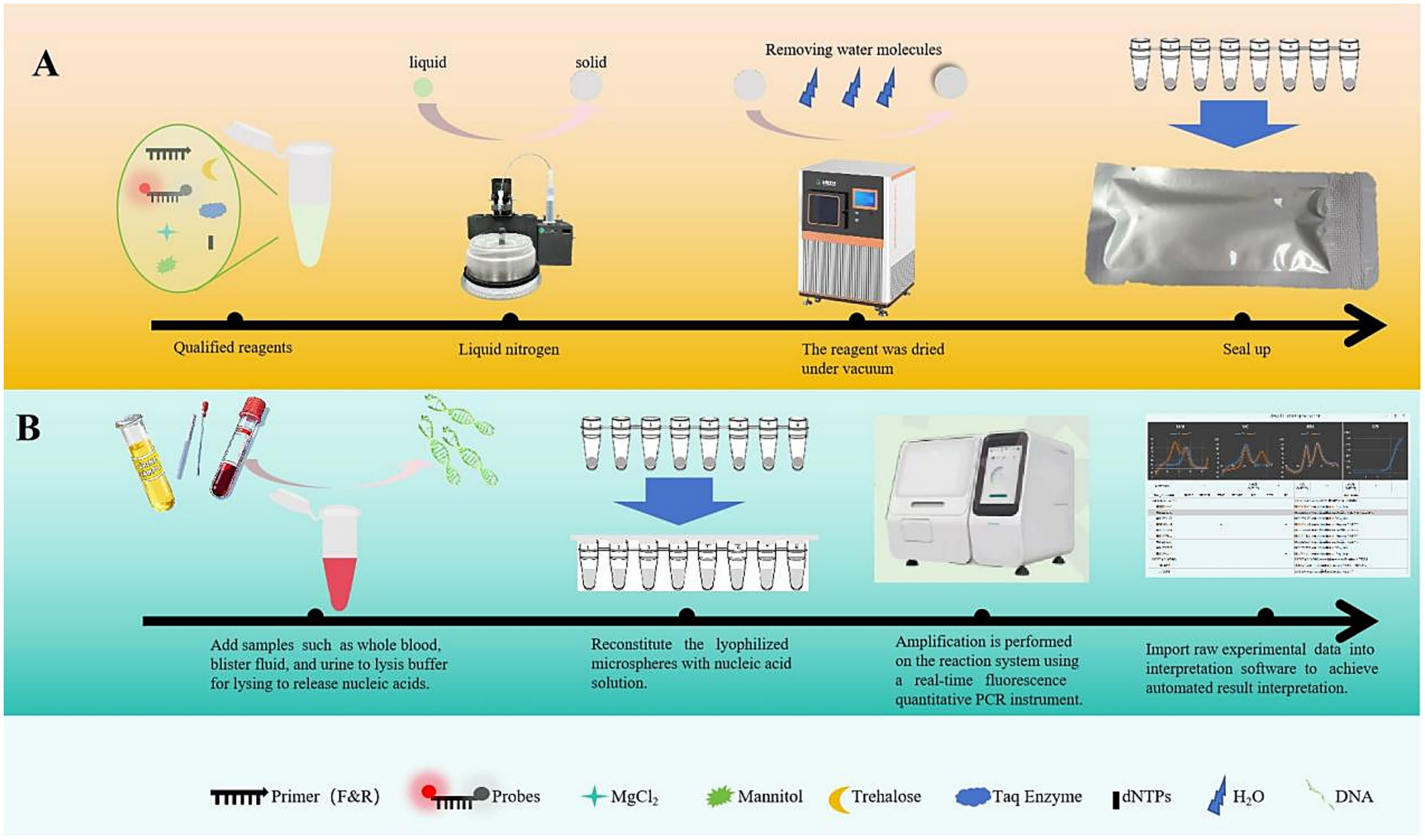
Schematic diagram of the integrated self-developed TORCHes detection system. The system integrates the entire process of sample pretreatment, multiplex detection, and result interpretation, consisting of two core components. **(A)** Lyophilized microsphere reagent preparation process (left to right): the optimized system undergoes liquid nitrogen treatment, vacuum drying to remove water molecules, and is then dispensed into lyophilized microspheres for sealed storage. This enables the integration of reaction components (primers/probes, enzymes, dNTPs, etc.) and cold-chain-independent transportation. **(B)** Sample detection process (left to right): samples (whole blood, blister fluid, urine, etc.) are lysed to release nucleic acids, reconstituted with lyophilized microspheres, subjected to PCR amplification, and finally analyzed using TORCHes-MCA v1.0 software for automated melting curve interpretation and pathogen typing result output.

Therefore, we aimed to develop a novel “Streamlined Integrated TORCHes Detection System.” The rationale of this system was to seamlessly integrate rapid chemical lysis (a manual sample processing step) for nucleic acid release, ready-to-use stabilized freeze-dried multiplex PCR master mix, and dedicated software for entirely automated, objective result interpretation (Figure 1). By combining simplified manual preparation with fully automated analysis, this integrated approach aimed to significantly streamline the workflow and reduce operator dependency. Our study does not aim to replace serological screening but rather provide a powerful adjunct tool. It is designed to facilitate the confirmation of active infection and enable precise pathogen subtyping in complex clinical situations where serology alone proves inadequate, ultimately aiming to enhance diagnostic confidence in TORCHes-related cases.

## Methods

### Study population and specimens

Patients presenting to the Affiliated Jinhua Hospital of Zhejiang University School of Medicine, Jinhua Maternal and Child Health Hospital, and Jinhua Municipal People’s Hospital between April 2024 and October 2025 with clinically suspected TORCHes infections (TOX, RV, HSV-I/II, EBV, or HCMV) were recruited. A total of 210 clinical specimens were included for validation, comprising 157 whole-blood samples, 17 urine samples, 13 herpes fluid samples, 6 cerebrospinal fluid samples, 5 amniotic fluid samples, and 12 swab samples (e.g., throat and urethral swabs). The study was approved by the hospital ethics committee (Research 2024-Ethical Review-71) and conducted in accordance with the Declaration of Helsinki and its later amendments.

### Rapid nucleic acid release and assay principle

A self-prepared lysis buffer (Table 1) was used for rapid, instrument-free nucleic acid release. Specimens were standardized to a 300-μL input volume for lysis. When body-fluid volume was <300 μL (e.g., herpes fluid, cerebrospinal fluid, and amniotic fluid), sterile normal saline was added to achieve a final volume of 300 μL. Swabs were placed in 500 μL of sterile physiological saline and agitated for 30 s, after which 300 μL of the eluate was used for processing. For lysis, 300 μL of the specimen (or processed eluate) was combined with 1,000 μL lysis buffer, mixed for 1 min, and briefly centrifuged (16,000 g, 10 s) to pellet debris; the supernatant was used as the nucleic acid-containing lysate for downstream amplification.

**Table 1:** Cell lysis solution formula.

Name	Concentration	Function
Tris-hydrochloride (Tris–HCl)	50 mM	Buffer
Sodium hydroxide (NaOH)	150 mM	Osmotic agent
Phenylmethylsulfonyl fluoride (PMSF)	1 mM	Protease inhibitor
Ethylenediaminetetraacetic acid (EDTA)	1 mM	Stabilizer
Nonidet P-40 (NP-40)	1% (v/v)	Detergent

The assay employed a probe-consumption readout integrated with multicolor melting curve analysis. During amplification, target-specific probes hybridized to complementary amplicon regions; in the presence of target, the 5′ nuclease activity of Taq polymerase hydrolyzed the bound probe during extension, preventing generation of its characteristic post-amplification melting signal. Accordingly, target positivity was defined by the disappearance (loss) of the expected melting peak for that probe, whereas target-negative samples retained intact probes that yielded distinct melting peaks, enabling objective calling based on predefined peak patterns across fluorescence channels.

### Target selection and multiplex design

Targets were selected based on diagnostic relevance, sequence conservation for robust amplification, and compatibility with multiplexing and subtype discrimination. TOX targeted the *B1* gene per WS/T 486-2015 (National Health and Family Planning Commission of the People’s Republic of China, 2015). RV targeted the *E1* gene due to its higher conservation relative to alternative structural gene options (e.g., *E2*), supporting stable detection across variants. For HSV-I/II, EBV, and HCMV, we followed the multiplex PCR strategy described by Xu et al. (2024), selecting UL30 (HSV-I/II), BKRF1 (EBV), and UL123 (HCMV). Leveraging sequence homology among these targets, multiplex detection and HSV subtyping were performed using a primer architecture consisting of a single universal upstream primer combined with two downstream primers for discriminatory amplification. An internal control was incorporated using a GAPDH-targeting probe (Cy5 at the 5′ end and BHQ3 at the 3′ end) to monitor overall process integrity. Sample validity was assessed based on both amplification behavior (Ct <38) and the presence of the expected internal-control melting peak, ensuring reliable detection and minimizing false-negative reporting.

Primers and probes were designed for stable multiplex amplification and channel-resolved melting analysis while minimizing peak overlap and cross-interactions; beacon fragments were incorporated at both the 5′ and 3′ ends of probes to fine-tune *T*_m_ separation. Oligonucleotides were synthesized commercially (Shanghai Shenggong Biotechnology Co., Ltd.). The sequences are provided in Table 2. Reaction composition was iteratively optimized using a commercial PCR buffer/enzyme mixture (Shanghai Janzy Biotechnology Co., Ltd.) by adjusting primer/probe concentrations and evaluating peak placement and separation across channels. The final optimized concentrations in the reaction system were as follows: 600 nM (1245F), 400 nM (4R), 400 nM (125R), 80 nM (HSV I-1P), 65 nM (HSV II-2P), 90 nM (EBV-4P), 70 nM (HCMV-5P), 400 nM (Tox F1), 400 nM (Tox R2), 90 nM (Tox P1), 500 nM (RV-F1), 500 nM (RV-R1), 100 nM (RV-P1), 200 nM (GAPDH-F1), 200 nM (GAPDH-R1), and 100 nM (GAPDH-P1).

**Table 2:** Sequencing primers and probes for TORCHes pathogenes.

Pathogen	Primer/Probe	Sequence (5′–3′)
Herpes virus	1245F	TCATCTACGGGGACACGGAC
	4R	AAACTTGCAGGCCGTCTTCC
	125R	TGCGCACCARATCCACG
	HSV I-1P	FAM-CCGCGCCATATTTGTGCTGTGCCGCGGCGCGG-BHQ1
	HSV II-2P	FAM-CGCGCGCGCATTTTCGTTTTGTGCCGCGGCGCGCGCGCG-BHQ1
	EBV-4P	VIC-CGCGCGCTGATATGTGGGGGTGCTGACGGACGGCAGCGCGCG-BHQ1
	HCMV-5P	VIC-CGCGCGCGCCCTCCACGTTACATCGGCAAAGTGGAGGGCGCGCGCG-BHQ1
*Toxoplasma gondii*	Tox-F1	GGTCGACTTCATGGGACGATAT
	Tox-R2	TCCGTCGTAATATCAGGCC
	Tox-P1	ROX-CGCGCGCGCCGCTGTCTGTCTAGGGCACGCGCGCGCG-BHQ2
Rubella virus	RV-F1	GATGAGGTTCTTGCCCCCG
	RV-R1	ACAACATCGCGCACTTCCC
	RV-P1	ROX-CCGCGCCCCGTCGGCAGTTGGGTAAGAGACCAGGCGCGG-BHQ2
GAPDH	GAPDH-F1	TCGCTCAGACACCATGGG
	GAPDH-R1	CTACGACTGCAAAGACCCG
	GAPDH-P1	CY5-TGAAGGTCGGAGTCAACGGGTG-BHQ3

### Freeze-dried microspheres and PCR/MMCA conditions

**Figure 2:**
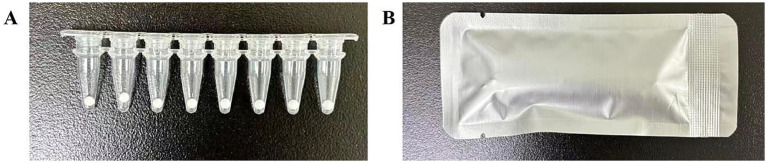
The finished product of freeze-dried microspheres. **(A)** Freeze-dried microspheres in an eight-tube strip. **(B)** The eight-tube strip containing the freeze-dried microspheres is depicted after sealing.

To eliminate cold-chain dependence and develop a ready-to-use multiplex format, reaction components were fabricated as freeze-dried microspheres using vacuum lyophilization. A lyoprotectant system consisting of trehalose (25% w/v), dithiothreitol (0.8 mol/L), bovine serum albumin (0.5% v/v), and mannitol (25% w/v) was incorporated to preserve enzyme activity and probe stability during drying and storage. Reaction premix aliquots (20 μL per microsphere) were dispensed into a cryogenic medium to form uniform spherical structures, followed by staged vacuum freeze-drying under controlled temperature and pressure conditions to ensure structural stability and functional preservation. After drying, microspheres were sealed in dry PCR eight-tube strips for ambient storage (Figure 2). Freeze-drying was performed by Shanghai Janzy Biotechnology Co., Ltd.

For detection, each reaction was initiated by rehydrating a single freeze-dried microsphere with 20 μL nucleic acid lysate supernatant. Amplification and melting analysis were performed on the SLAN-96P platform (HONGSHI, China) using the following program: 50 °C × 10 min; 95 °C × 5 min (1 cycle); 95 °C × 10 s and 60 °C × 40 s (40 cycles); followed by melting at 95 °C × 10 s and 20–60 °C at 0.03 °C/s (1 cycle). Multi-channel melting curves were generated automatically for downstream interpretation.

### Reference materials and analytical validation

Synthetic plasmids containing each target were produced commercially (Shanghai Shenggong Biotechnology Co., Ltd.) and used as reference materials. Plasmid concentrations were determined using an aminobenzyl-factor quantitative detection kit (PCR method). A standard curve was generated from serial dilutions of kit standards using qPCR measurements targeting the shared ampicillin resistance gene. Plasmid Ct values were interpolated from the regression equation to obtain concentrations for subsequent LoD, stability, and interference studies.

Analytical sensitivity, specificity, precision, stability, and interference tolerance were evaluated in a predefined framework. For sensitivity, plasmid standards for each pathogen were serially diluted in pooled pathogen-negative human serum to generate graded concentrations and processed using the same lysis and amplification workflow as clinical specimens; testing was performed in 20 replicates per concentration level, and LoD was defined as the lowest concentration achieving a detection rate ≥95%. To corroborate performance in real specimens, 20 clinically confirmed positive samples per pathogen were quantified/assigned concentrations using commercial assays (described below), adjusted to the target concentration series, and re-tested using the same workflow; LoD confirmation required ≥95% positivity (19/20) at the proposed threshold. Specificity was examined using clinically confirmed TORCHes-positive samples (HCMV, TOX, RV, HSV-I/II, EBV), samples positive for other pathogens (HIV, syphilis, HBV, HCV, *Neisseria gonorrhoeae*, *Chlamydia trachomatis*, and HPV), and DNA from healthy donors to assess cross-reactivity and non-specific signals. Precision was assessed based on reproducibility of *T*_m_ values after rehydrating microspheres with deionized water and directly performing melting analysis. Intra- and inter-batch precision were evaluated across five production batches (eight replicates per batch) and expressed as CVs for each target peak. Accelerated stability testing was performed by storing microspheres at 37 °C and evaluating performance at 6, 9, and 12 months using plasmids at low (LoD), medium (5 × LoD), and high (10 × LoD) concentrations (20 replicates per level); stability was considered acceptable if detection remained 100% at medium/high concentrations and ≥95% at the LoD level throughout testing.

Interference tolerance was assessed by spiking endogenous interferents (hemoglobin ≤5 g/L, triglycerides ≤20 mmol/L, IgG/IgM ≤50 g/L) and exogenous interferents (heparin ≤100 IU/mL, acyclovir/ganciclovir ≤1 μg/mL) into negative serum or whole-blood lysate matrices. Pathogen plasmids were added at 3 × LoD to generate interference-positive samples; interference-free positive controls, interference-matrix negative controls, and water blanks were included, each in triplicate, and all samples were processed using the established workflow. Resistance to interference was defined as correct positivity in interference-positive samples and minimal *T*_m_ deviation in interference-negative samples (≤1 °C vs. non-interference negative controls).

### Clinical comparison, discrepancy adjudication, and automated interpretation

Clinical performance was assessed by parallel testing of all 210 suspected clinical samples using the microsphere assay and commercial single-target qPCR reagents as comparators, including TOX and RV kits (Janzy Biotechnology Co., Ltd., Shanghai), HCMV and EBV kits (Shengxiang Biotechnology Co., Ltd., Hunan), and an HSV-I/II typing kit (PCR-fluorescence probe method; Prologg Bioproducts Co., Ltd., Shanghai). Discordant results between methods were adjudicated by Sanger sequencing using the primer sequences listed in Table 2.

To standardize melting-curve interpretation and eliminate subjectivity, TORCHes-MCA v1.0 was developed in Python 3.10 using NumPy, SciPy, and PyQt5. The software converted raw melting curves via a negative first-order derivative transformation (−dF/dT), identified candidate peaks, and applied Gaussian fitting $G(T)=A\cdot e^{-\frac{{\left(T-\mu \right)}^{2}}{2\sigma^{2}}}$ (*μ* as *T*_m_; *σ* as peak width) optimized by least squares to resolve adjacent peaks. Pathogen calls were produced by matching fitted peaks against channel-specific *T*_m_ thresholds, and outputs included pathogen typing and internal control results. Input files were supported from SLAN-96P/S and 7500 exports (Ct and melting peak data in Excel format). A quality-control module denoted “Extraction failed” when the internal control peak was absent or Ct >38. Baseline correction was implemented to reduce background noise. Multiprocessing enabled batch processing, and a fault-tolerant mechanism triggered secondary fitting when fit confidence was insufficient (*R*^2^ < 0.95) to prompt manual review.

### Statistical analysis

Agreement between methods was assessed using Cohen’s Kappa (SPSSAU online analysis platform). Kappa values were interpreted as follows: 0.8–1.0, strong agreement; 0.6–0.8, relatively strong; 0.4–0.6, moderate; 0.2–0.4, fair; < 0.2, poor.

## Results

### Establishment of the concentration of plasmid standards

**Figure 3:**
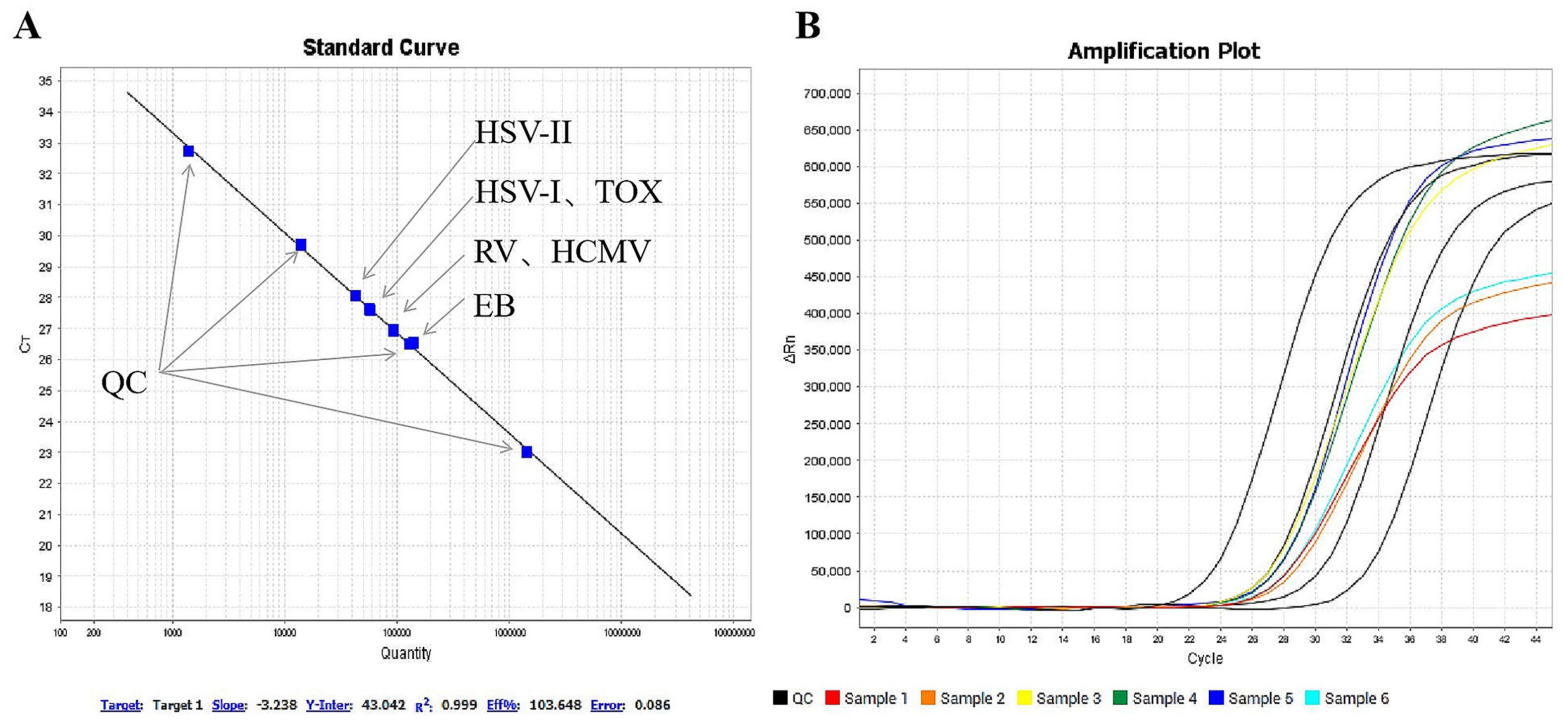
Establishment of concentrations for six plasmid standards. **(A)** Standard curve, *X*-axis: concentration; *Y*-axis: Ct value; virus/pathogen labels: HSV-I, TOX, RV, HCMV, EB, HSV-II; overlapping points due to similar concentrations are marked. **(B)** PCR amplification curve, Black = Standard substance, Red = HSV-I, Orange = HSV-II, Yellow = EBV, Green = HCMV, Blue = RV, Cyan = TOX.

The concentrations of the six synthetic plasmid standards, as determined by the amino benzyl factor quantitative kit, were as follows: HSV-I, 49,339 copies/mL; HSV-II, 37,329 copies/mL; EBV, 122,857 copies/mL; HCMV, 91,491 copies/mL; RV, 89,144 copies/mL; TOX, 51,301 copies/mL. The standard curve exhibited excellent linearity with a correlation coefficient (*R*^2^) of 0.998 (Figure 3).

### Single-tube multiplex detection performance

**Figure 4:**
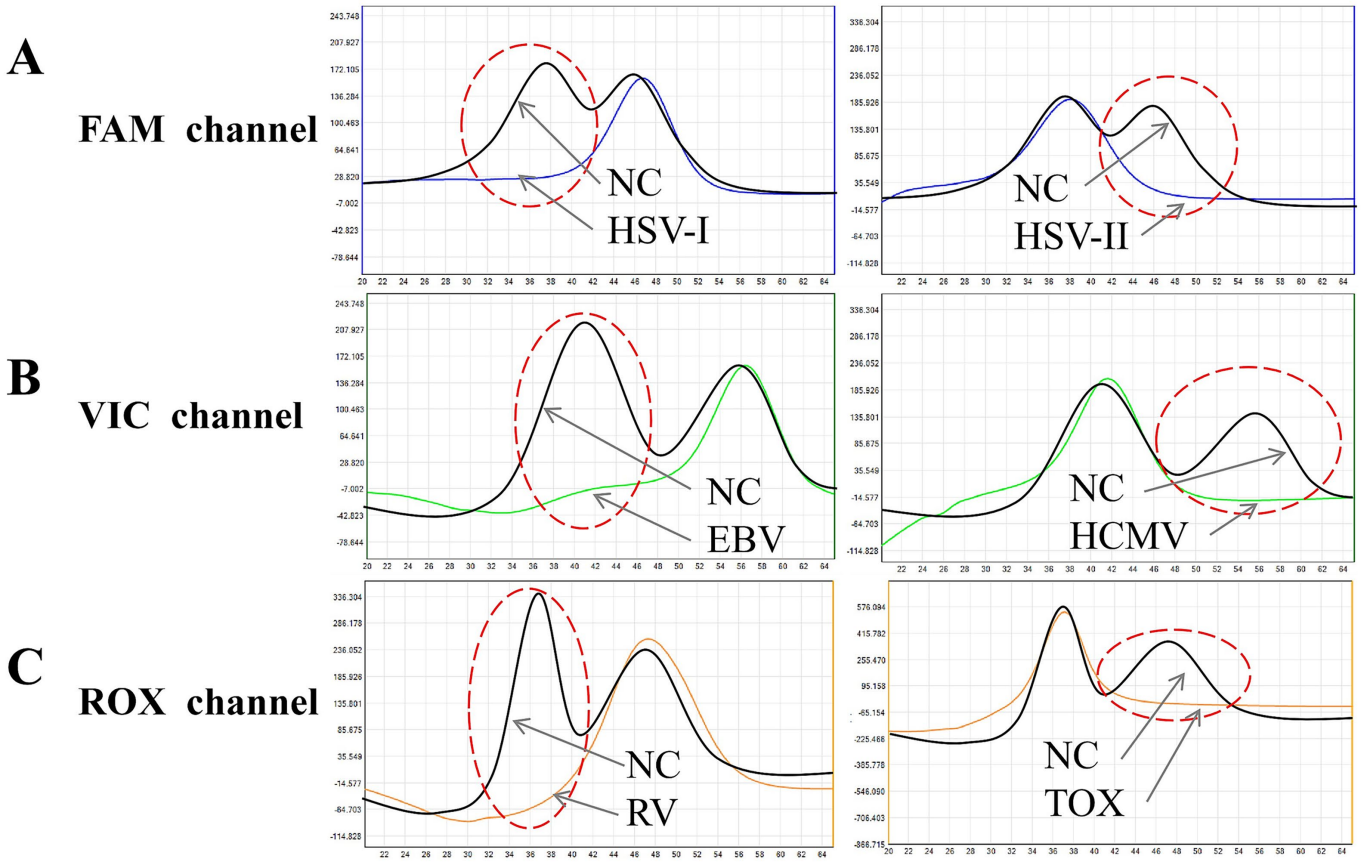
Melting curve analysis of TORCH pathogens in different fluorescence channels. **(A)** FAM channel (blue curve): HSV-I (first peak, 36.8 ± 1 °C) and HSV-II (second peak, 46.2 ± 1 °C). **(B)** VIC channel (green curve): EBV (first peak, 40.8 ± 1 °C) and HCMV (second peak, 55.8 ± 1 °C). **(C)** ROX channel (orange curve): RV (first peak, 37.0 ± 1 °C) and TOX (second peak, 46.2 ± 1 °C). Abscissa: temperature; ordinate: fluorescence value change rate. Black curves represent negative controls (NCs); red dashed circles indicate the peak disappearance of positive samples.

The freeze-dried microsphere-based MMCA assay enabled simultaneous differentiation of all six TORCHes pathogens within a single reaction. Representative melting curve analyses are shown in Figure 4. In the FAM channel, HSV-I and HSV-II yielded distinct melting peaks at 36.8 ± 1 °C and 46.2 ± 1 °C, respectively. In the VIC channel, EBV and HCMV were identified by peaks at 40.8 ± 1 °C and 55.8 ± 1 °C, respectively. In the ROX channel, RV and TOX were distinguished by peaks at 37.0 ± 1 °C and 46.2 ± 1 °C, respectively. A positive result was indicated by the disappearance of the corresponding pathogen-specific peak.

### Sensitivity and specificity verification

**Figure 5:**
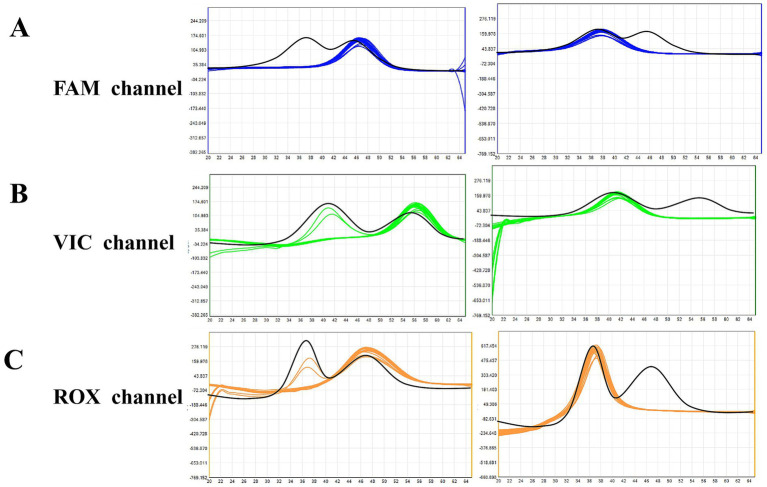
Repeatability test results of plasmid standards at 200 copies/mL. **(A)** FAM channel (blue curve): HSV-I (detection rate = 100%) and HSV-II (detection rate = 100%). **(B)** VIC channel (green curve): EBV (detection rate = 90%) and HCMV (detection rate = 100%). **(C)** ROX channel (orange curve): RV (detection rate = 90%) and TOX (detection rate = 100%). Abscissa: temperature; ordinate: fluorescence value change rate. Black curves represent negative controls (NCs); red-dashed circles indicate target peaks.

The LoDs were 200 copies/mL for HSV-I, HSV-II, HCMV, and TOX, and 500 copies/mL for EBV and RV (Figure 5 and Table 3). These LoDs were confirmed using 20 clinical positive samples for each pathogen, with detection rates of ≥95% at the respective LoD concentrations. Specificity testing showed that all TORCHes-positive samples were correctly detected and accurately typed. In contrast, samples positive for other pathogens and DNA samples from healthy donors were negative (Supplementary Table 1).

**Table 3:** Positive detection rates of serially diluted clinical samples.

Clinical samples concentration (copies/mL)	Relevance ratio					
	HSV-I	HSV-II	EBV	HCMV	RV	TOX
1,000	100%	100%	100%	100%	100%	100%
500	100%	100%	100%	100%	100%	100%
200	100%	100%	90%	100%	90%	100%
100	0	0	0	0	0	0

### Precision and stability verification

**Figure 6:**
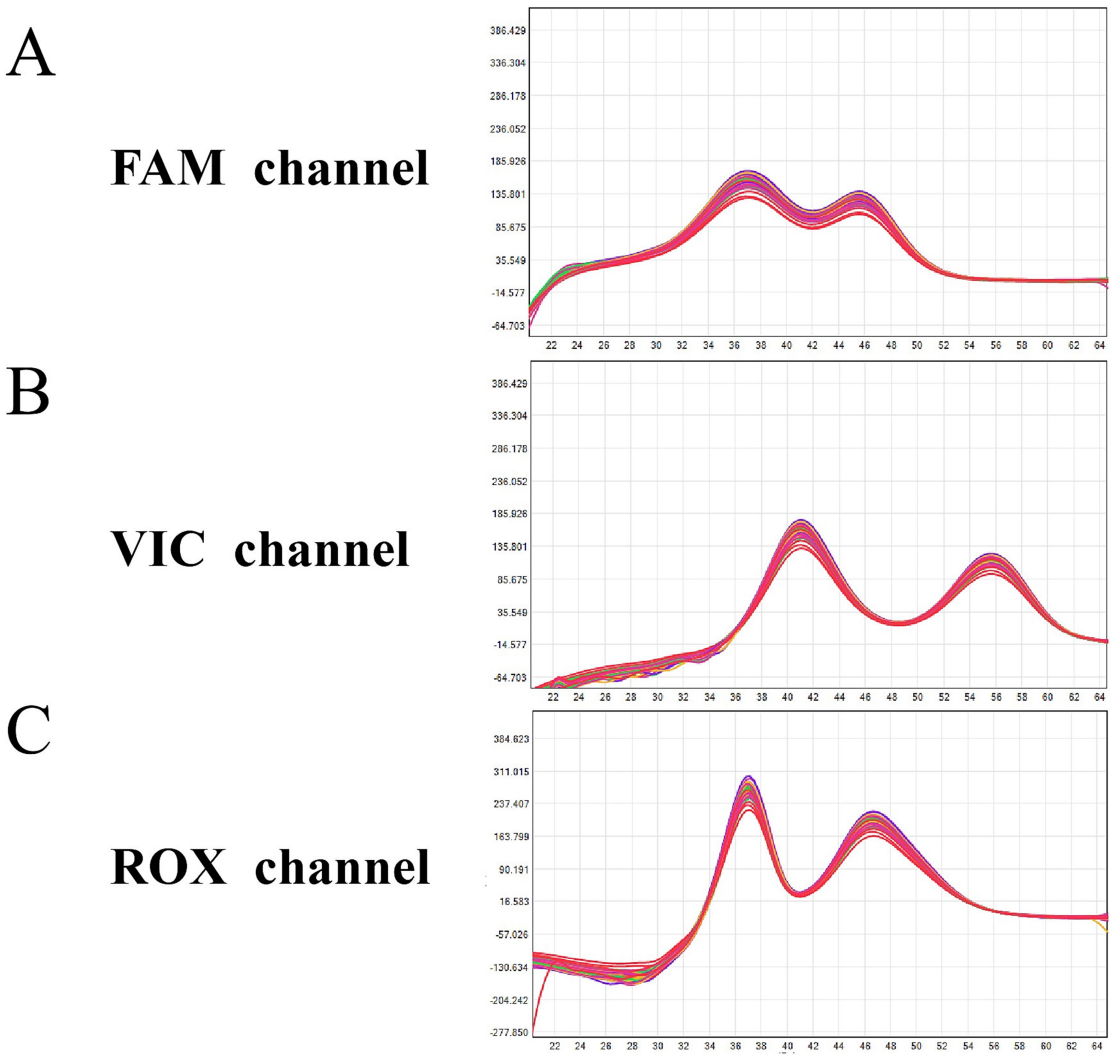
Precision validation melt curves of five lyophilized microsphere batches. **(A)** FAM channel (HSV-I/HSV-II). **(B)** VIC channel (EBV/HCMV). **(C)** ROX channel (RV/TOX). Light green, yellow, orange, gray, and red curves represent batches 1–5, respectively (eight replicates per batch). Black curves denote DD-H_2_O negative controls. *T*_m_ values of all targets showed minimal intra-batch and inter-batch variation (detailed in Table 4).

**Figure 7:**
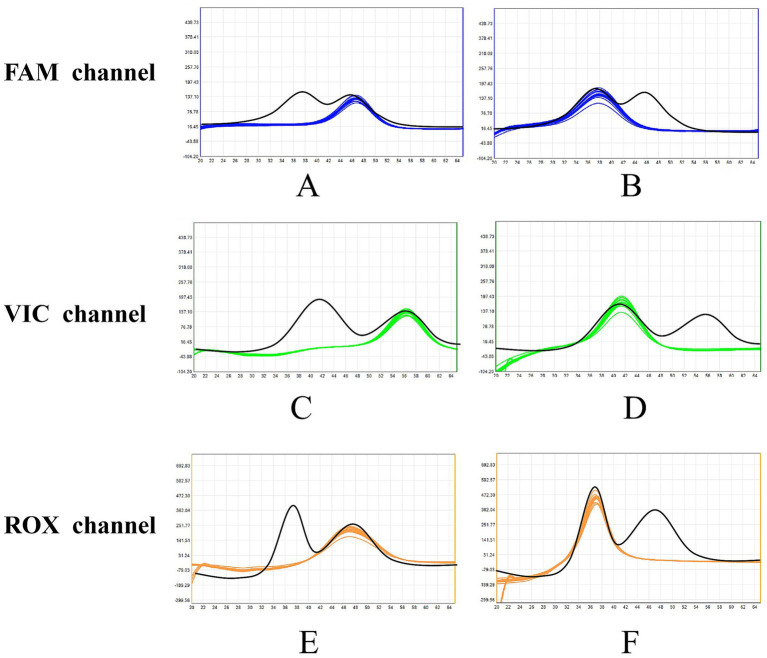
Stability verification melt curves of lyophilized microspheres at LoD level after 12 months of storage at 37 °C. Testing was conducted against six plasmid standards at their respective LoD levels (*n* = 20 replicates). **(A)** HSV-I (200 copies/mL, FAM channel, blue curve). **(B)** HSV-II (200 copies/mL, FAM channel, blue curve). **(C)** EBV (500 copies/mL, VIC channel, green curve). **(D)** HCMV (200 copies/mL, VIC channel, green curve). **(E)** RV (500 copies/mL, ROX channel, orange curve). **(F)** TOX (200 copies/mL, ROX channel, orange curve). Abscissa: temperature; ordinate: fluorescence value change rate. Black curves represent negative controls (NCs); red dashed circles indicate target peaks. All targets showed 100% detection rate (consistent with stability criteria: high/medium concentration 100%, low concentration ≥95%).

The intra-batch and inter-batch CVs for the *T*_m_ values of all targets ranged from 0.03 to 0.18% (Figure 6 and Table 4). Under accelerated storage at 37 °C, the freeze-dried microspheres maintained performance for at least 12 months, with detection rates remaining 100% for high (10 × LoD) and medium (5 × LoD) concentration samples. At the LoD level, detection rates met the acceptance criterion of ≥95% across the entire testing period (Figure 7). The finished product (see Figure 2) was designed for long-term storage at room temperature, eliminating the need for cold-chain logistics.

**Table 4:** Coefficient of variation (CV) of *T*_m_ values across five lyophilized microsphere batches.

Batches	HSV-I	HSV-II	EBV	CMV	RV	TOX
1	36.83	46.11	40.78	55.81	36.98	46.25
2	36.67	46.14	40.83	55.85	37.01	46.33
3	36.86	46.15	40.84	55.81	36.96	46.19
4	36.78	46.13	40.84	55.84	36.97	46.24
5	36.80	46.22	40.80	55.80	36.96	46.20
CV%	0.18	0.08	0.05	0.03	0.06	0.11

### Anti-interference study

Common endogenous (hemoglobin ≤5 g/L, triglycerides ≤20 mmol/L, immunoglobulins ≤50 g/L) and exogenous (heparin ≤100 IU/mL, acyclovir/ganciclovir ≤1 μg/mL) interferents did not affect assay performance. All positive samples containing interferents were correctly detected, and *T*_m_ values for negative samples containing interferents deviated by less than 0.5 °C from controls (Table 5 and Supplementary Figure S5).

**Table 5:** *T*_m_ value shifts of target melting peaks in the presence of interferents.

Interfering substances	Concentration	*T*_m_ shift (°C)	False positive rate
Hemoglobin	5 g/L	≤0.3	0%
Triglycerides	20 mmol/L	≤0.2	0%
Immunoglobulin IgG/IgM	50 g/L	≤0.4	0%
Heparin	100 IU/mL	≤0.3	0%
Acyclovir	1 μg/mL	≤0.2	0%

### Comparison of methodology

Among the 210 clinical samples, Kappa values for each pathogen ranged from 0.965 to 1.000, indicating high agreement with the comparator assay (Table 6). TOX results were completely consistent (Kappa = 1.000). The Kappa values for HSV-I, HSV-II, EBV, HCMV, and RV were 0.986, 0.967, 0.986, 0.984, and 0.965, respectively. Results for 16 mixed-infection samples were concordant between methods; the most frequent co-detections were EBV and RV (68.75%, 11/16). Seven discordant cases were resolved by Sanger sequencing, and in all instances, the results generated by this method were confirmed as correct (Supplementary Figure S6). Notably, across diverse clinical sample types (whole blood, urine, herpes fluid, cerebrospinal fluid, amniotic fluid, and swabs), our method maintained high overall agreement (96.67%, 203/210) with fluorescent PCR, with 100% concordance for herpes fluid, cerebrospinal fluid, and amniotic fluid, and >91% for blood, urine, and swabs (Supplementary Table 2).

**Table 6:** Agreement analysis between the developed method and reference method for 210 clinical samples.

New TORCHes typing method		Fluorescent PCR method analysis			
Pathogen types		+	−	Kappa values	95% CI
HSV-I	+	41	0	0.986	[0.958, 1.000]
	−	1	168		
HSV-II	+	34	0	0.967	[0.922, 1.000]
	−	2	174		
EBV	+	43	0	0.986	[0.959, 1.000]
	−	1	166		
CMV	+	37	1	0.984	[0.954, 1.000]
	−	0	172		
RV	+	32	1	0.965	[0.918, 1.000]
	−	1	176		
TOX	+	12	0	1.000	[1.000, 1.000]
	−	0	198		

### Melting curve interpretation software

**Figure 8:**
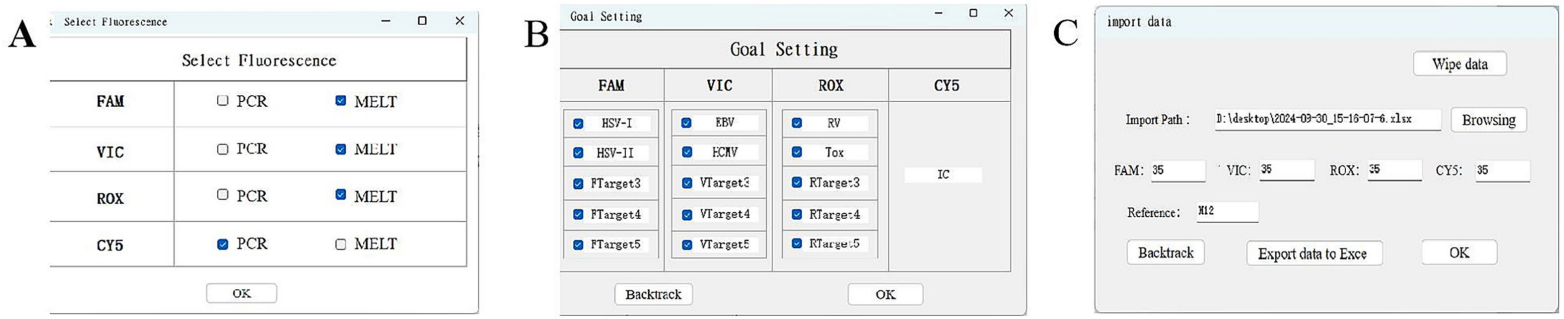
TORCHes-MCA v1.0 software outputs after data export. **(A,B)** Selection of channels and corresponding detection targets for experiment. **(C)** The exported data were imported into the interpretation software, and the hole position of the negative control was selected by referring to the hole number. After completion of these settings, the “Import data” option was selected to automatically identify the experimental results.

**Figure 9:**
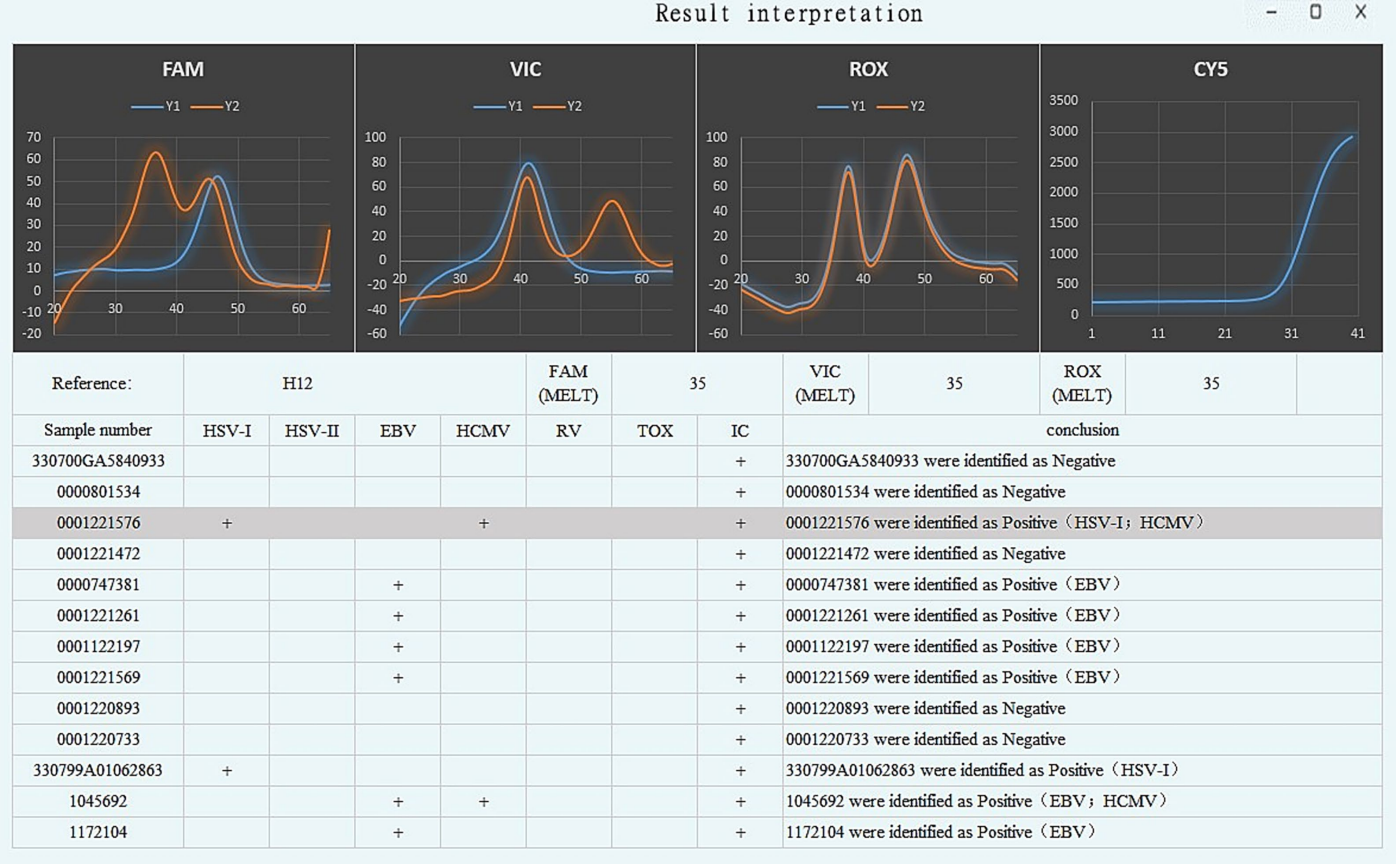
Example of TORCHes-MCA v1.0 software output results. The software interface consists of two main sections: the top panel displays detection curves for four fluorescent channels (FAM, VIC, ROX, and CY5), where orange lines represent sample detection signals and blue lines represent negative controls/internal controls; the bottom panel shows a sample result table. The gray highlighted sample (No. 0001221576) was identified as positive for HSV-I and HCMV. Specifically, the blue curve in the CY5 channel represents the internal control (for quality control), corresponding to the “+” symbol in the “IC” column of the table, which indicates a valid internal control. Curves in the FAM/VIC/ROX channels correspond to pathogen typing assays, where “+” symbols in target columns (e.g., HSV-I, HSV-II, EBV) indicate positive pathogens. The final result for each sample is summarized in the “conclusion” column.

TORCHes-MCA v1.0 was used to automatically analyze raw melting curve data and generate result outputs, with the operational workflow shown in Figure 8. The software automatically reported pathogen typing results and internal control Ct values (Figure 9) and flagged “extraction failure” when the internal standard was abnormal (peak missing or Ct >38). Using the dataset from the 210 clinical samples processed by cell lysate and tested with freeze-dried microspheres, the results showed that the software outputs were fully concordant with manual interpretation, yielding a coincidence rate of 100%.

## Discussion

At present, the clinical detection of TORCHes pathogens mainly relies on ELISA, chemiluminescence immunoassay (CLIA) and single fluorescent quantitative PCR technology. Despite the high sensitivity of ELISA and CLIA, serological methods have a high false positive rate due to cross-reaction of antibodies. One study of prepregnant women showed that ELISA IgM had a false positive rate of 67.3% (Paquet and Yudin, 2018). CLIA is limited by technical bottlenecks such as short luminescence time of signal molecules (Sun et al., 2021; Kemppainen et al., 2018; Cook et al., 2021). Although single fluorescent quantitative PCR can achieve early nucleic acid diagnosis, it has problems such as low throughput (multiple tubes of reaction are required to cover six pathogens) and liquid reagents rely on strict cold chain storage and transportation (−20 °C), which means its application in remote areas is limited (Vinjé, 2015). The shortcomings in operation complexity, stability and accuracy of these methods severely limit the efficiency of large-scale screening.

In this study, a streamlined technical system of “nucleic acid extraction of cell lysate—multiple detection of freeze-dried microspheres—intelligent interpretation of melting curve” was successfully constructed, which effectively solved the three bottlenecks of traditional detection, such as complicated operation, poor stability and subjectivity of interpretation: (1) Operation convenience: By optimizing the components, the lysate efficiently released nucleic acids within 1 min and was stable in hemolytic/lipid blood samples, significantly simplifying the pretreatment; (2) Stability and integration: the freeze-dried microspheres were protected by trehalose-DTT system and stable at 37 °C for 1 year. Based on the freeze-dried microspheres and MMCA technology, six TORCHes pathogens could be detected simultaneously in a single tube, completely eliminating the dependence on cold chain; (3) Intelligent interpretation: the matching TORCHes-MCA v1.0 software improved the typing efficiency through algorithm optimization and eliminates manual interpretation errors. The system showed excellent performance including high sensitivity (LoD = 200 copies/mL for HSV-I/II/HCMV/TOX, LoD = 500 copies/mL for EBV/RV), high specificity, good precision (both intra-batch and inter-batch CV <5%) and strong tolerance to specific interferences. In validating 210 clinical samples, our method showed high consistency with control qPCR (Kappa 0.965–1.000; 100% coincidence) and strong agreement across diverse specimen types, including 100% concordance for herpes fluid, CSF, and amniotic fluid, supporting broad clinical utility. This indicated that the closed-loop system constructed in this study significantly improved the detection convenience, stability and multi-target detection ability, and provided an efficient and reliable new tool for high-throughput TORCHes pathogen identification.

The core of this stability breakthrough lied in the innovative application of vacuum freeze-drying technology and its successful integration with multiple detection systems. By effectively removing water, this technology significantly improved the stability of reagents and reduced the dependence of storage and transportation temperature, thereby prolonging the shelf life while maintaining the reaction activity and sensitivity. The key breakthrough was that the complex multiple primer and probe system for six TORCHes pathogens was successfully lyophilized into ready-to-use microspheres, eliminating the tedious and error-prone operation steps of traditional liquid reagents such as frequent preparation, packaging, and premix. Through the optimization of protective agents (such as trehalose and DTT), the challenge of maintaining the multiple amplification efficiency and melting curve accuracy after freeze-drying was resolved. Thus, freeze-dried microspheres not only showed excellent performance (HSV-I/II/HCMV/TOX LoD = 200 copies/mL) comparable to or even better than single-weight qPCR (Shanghai ProMag Biotechnology Co., Ltd., 2023) but also maintained 100% detection of LoD-concentration plasmids after 1 year of storage at 37 °C. Notably, the absence of a cold chain requirement significantly enhances operational efficiency. It transformed the testing process into a more streamlined and robust procedure, ensuring reliable performance in daily laboratory practice.

Notably, our self-prepared lysate nucleic acid extraction protocol, optimized through the synergistic effect of osmotic regulator and detergent, enabled efficient nucleic acid release from a wide range of sample types—including whole blood, herpes fluid, urine, cerebrospinal fluid, amniotic fluid, and multiple swabs—in under 1 min, without relying on commercial extraction instruments. This one-step lysis protocol was designed for rapid nucleic acid release rather than purification and concentration. While commercial extraction kits may provide higher nucleic acid yields via multi-step enrichment processes, our simplified approach delivered nucleic acid of sufficient quality and quantity, as evidenced by the high analytical sensitivity (LoD: 200–500 copies/mL), robust inhibitor tolerance, and excellent clinical agreement achieved in this study. Compared to traditional magnetic bead-based nucleic acid extraction kits—which typically require six core manual steps (lysis, binding, two washes, elution, and collection)—our one-step direct lysis protocol reduced the key processing operations by approximately 83% (5 out of 6 steps). This substantial simplification significantly reduced hands-on time and lowered the risk of cross-contamination, rendering the method particularly suitable for settings prioritizing workflow simplicity and speed over maximum nucleic acid recovery. Such a streamlined, instrument-free workflow is especially well-suited for primary healthcare settings with limited access to specialized laboratory equipment, enhancing the feasibility of molecular testing in resource-constrained environments. At the same time, the designed freeze-dried microspheres exhibited excellent platform compatibility, supporting both crude nucleic acid from our direct lysis and high-purity nucleic acid extracted by commercial kits, thereby providing users with simple and high-sensitivity dual-track detection paths. This flexibility can accommodates resource differences across healthcare institutions—enabling primary hospitals to perform “sample direct detection” while allowing tertiary hospitals to integrate with existing automated platforms to improve throughput, thus offering technical support for hierarchical healthcare systems.

In terms of primer design, we adopted the strategy of “single universal upstream primer + specific downstream primer” (applied to HSV-I/II, EBV and HCMV) to accurately screen conserved gene regions, thereby reducing the total number of primers from conventional 24 to 12 (a 50% reduction) (Abla and Mehanna, 2022). This simple architecture effectively reduced the secondary structure interference and cross-reaction risk between primers, improved the amplification efficiency of multiplex PCR, and provided a key guarantee for the system to achieve high sensitivity (HSV-I/II LoD = 200 copies/mL) and stability. This strategy provided an optimized paradigm for the development of primer design for higher throughput multiplex pathogen nucleic acid detection.

In order to completely eliminate manual interpretation errors and improve efficiency, the TORCHes-MCA v1.0 software was developed to integrate the core algorithm and intelligent quality control function. Based on negative first derivative transformation and Gaussian fitting algorithm, the melting peak was accurately identified (temperature difference resolution ≤0.5 °C). Clinical verification showed that the coincidence rate with manual interpretation was 100%, which ensured the objectivity of typing. The software incorporated a dynamic background calibration function to effectively eliminate interference from factors, such as hemolysis (≤5 g/L Hb) and antibiotics, (acyclovir ≤1 μg/mL), thereby ensuring zero false-positive results. Simultaneously, an internal control mechanism flagged extraction failure in real time when the internal standard GAPDH melting peak was missing or Ct >38, thereby avoiding the risk of false negative. The software worked seamlessly with the freeze-dried microspheres to form a closed loop of “reagent-data-algorithm,” and the standardized output (pathogen typing) could be directly linked to the LIS system. This design realized the automatic process from “sample in” to “result out,” which was especially suitable for large-scale screening in resource-poor settings and significantly improved the throughput and reliability of test results. While our system is qualitative in nature (reporting presence/absence of pathogen nucleic acid), it provides direct evidence of active viral replication. By detecting the pathogen’s genetic material in clinical samples, it directly confirms the presence of the infectious agent itself, circumventing the inherent lag and ambiguity of indirect serological markers. Its result, when integrated into the clinical diagnostic pathway diagnostic pathway recommended by the Expert Consensus, is pivotal for concluding whether an active infection is occurring, especially in clarifying equivocal serology and determining the HSV subtype.

This study successfully established a streamlined technology system integrating “cell lysate extraction, freeze-dried microsphere multiplex detection, and intelligent interpretation software.” This integrated system demonstrated significant advantages in terms of procedural simplicity, enhanced reagent stability, and objective result reporting, offering a practical alternative for decentralized testing environments, particularly those lacking stable cold-chain infrastructure. However, the generalizability of the system across larger populations and diverse sample variations needs further validation through multicenter studies, and the long-term practical effectiveness and cost-efficiency require additional evaluation in real-world clinical practice.

## Limitations

This study has some limitations. First, the clinical validation, while demonstrating excellent agreement with reference methods, was performed primarily at a single center with a sample size that yielded limited positive cases for some lower-prevalence pathogens (e.g., TOX). Second, the uneven distribution of sample types means that performance in certain matrices (e.g., CSF, amniotic fluid) was assessed in a smaller number of specimens. Future multi-center studies with larger, prospectively enrolled cohorts are warranted to confirm the robustness and clinical utility of this system across diverse populations and specimen types, and to obtain more precise performance estimates for each individual pathogen.

## Conclusion

This study successfully established a streamlined, integrated TORCHes detection system featuring “one-minute lysate-based nucleic acid liberation”, “cold chain-independent multiplex PCR via freeze-dried microspheres”, and “algorithm-driven interpretation.” This system effectively overcame the key limitations of traditional molecular methods, namely operational complexity, reagent instability, and interpretive subjectivity. With its advantages of extreme simplicity, high stability, and excellent multiplexing capability, the system provides an efficient and reliable novel tool for large-scale TORCHes screening, demonstrating significant potential for application in primary healthcare settings.

## Data Availability

The original contributions presented in the study are included in the article/Supplementary material, further inquiries can be directed to the corresponding authors.
