# Supplementary material for: A streamlined integrated system integrating lysate release, freeze-dried reagents for multiplex polymerase chain reaction, and intelligent analysis for TORCHes pathogen identification
Source: Front Microbiol. 2026 Mar 24;17:1788209. doi: 10.3389/fmicb.2026.1788209 (PMC13053493; doi:10.3389/fmicb.2026.1788209)
Supplement: Supplementary file 1 [file Table_1.DOC]

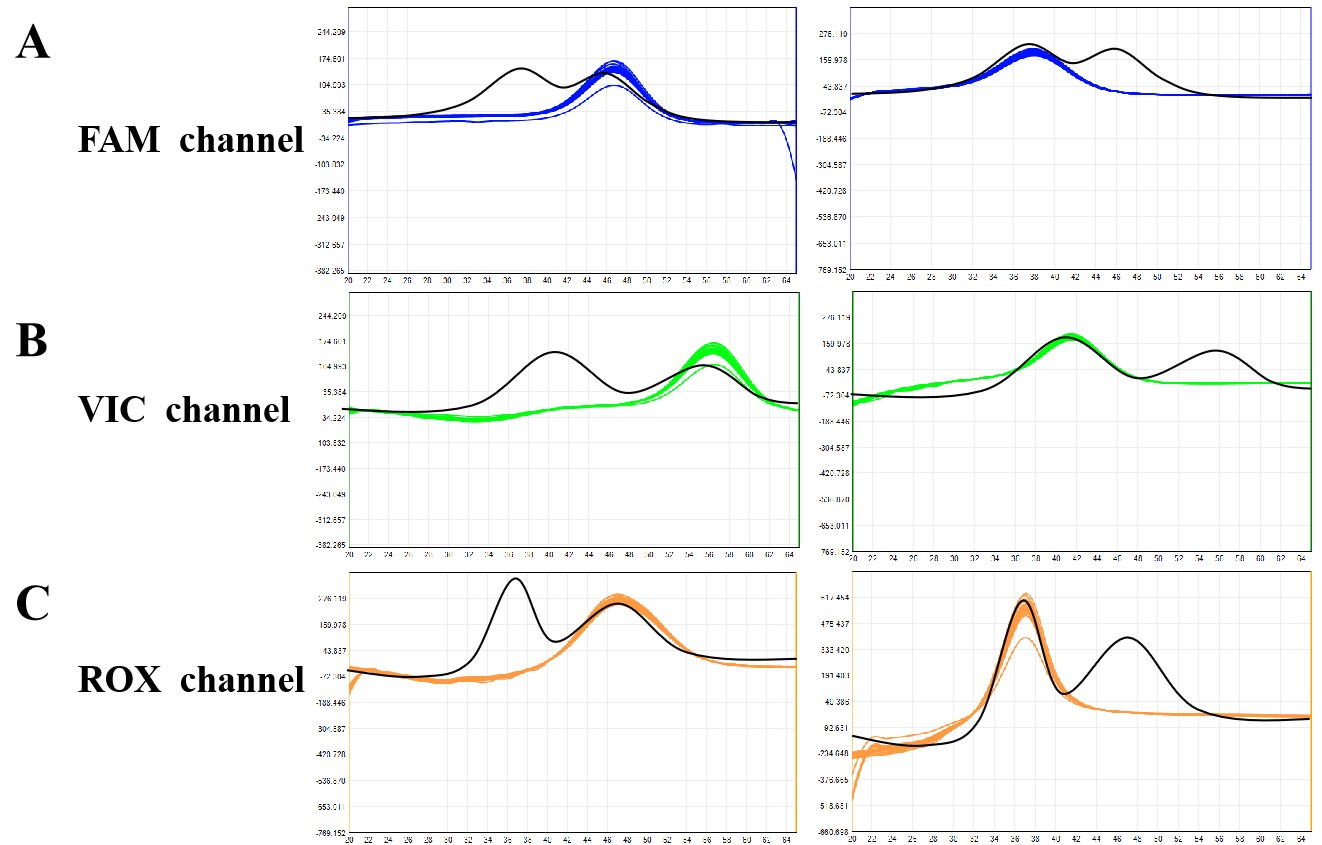


**Figure S1: Repeatability test results of plasmid standards at 1000 copies/ml.** (A) FAM channel (blue curve): HSV-I and HSV-II. (B) VIC channel (green curve): EBV and HCMV. (C) ROX channel (orange curve): RV and TOX. All 6 pathogens were stably detected (20/20 replicates). Abscissa: Temperature; Ordinate: Fluorescence value change rate. Black curves represent negative controls (NCs).


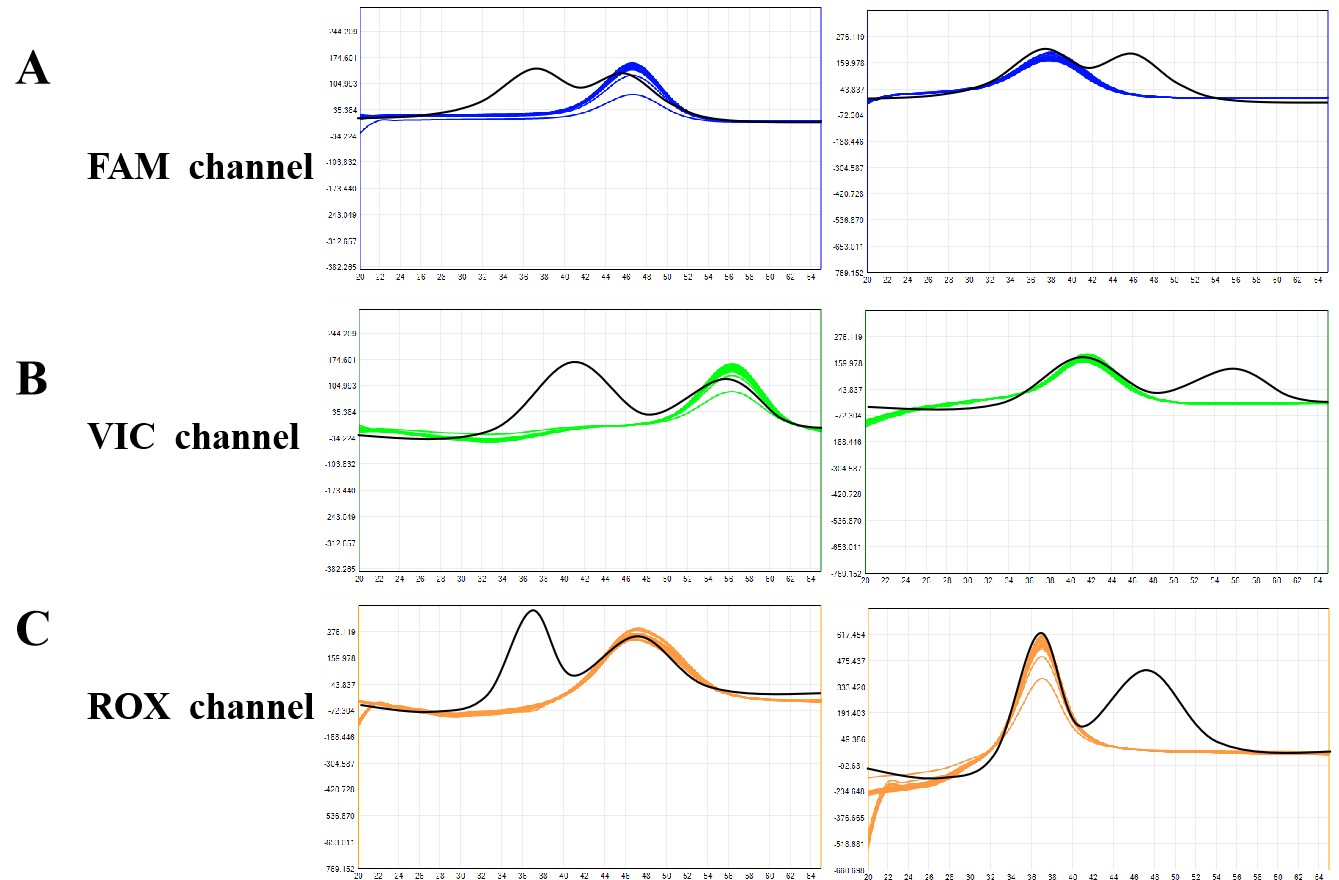


**Figure S2: Repeatability test results of plasmid standards at 500 copies/ml.** (A) FAM channel (blue curve): HSV-I and HSV-II. (B) VIC channel (green curve): EBV and HCMV. (C) ROX channel (orange curve): RV and TOX. All 6 pathogens were stably detected (20/20 replicates). Abscissa: Temperature; Ordinate: Fluorescence value change rate. Black curves represent negative controls (NCs).


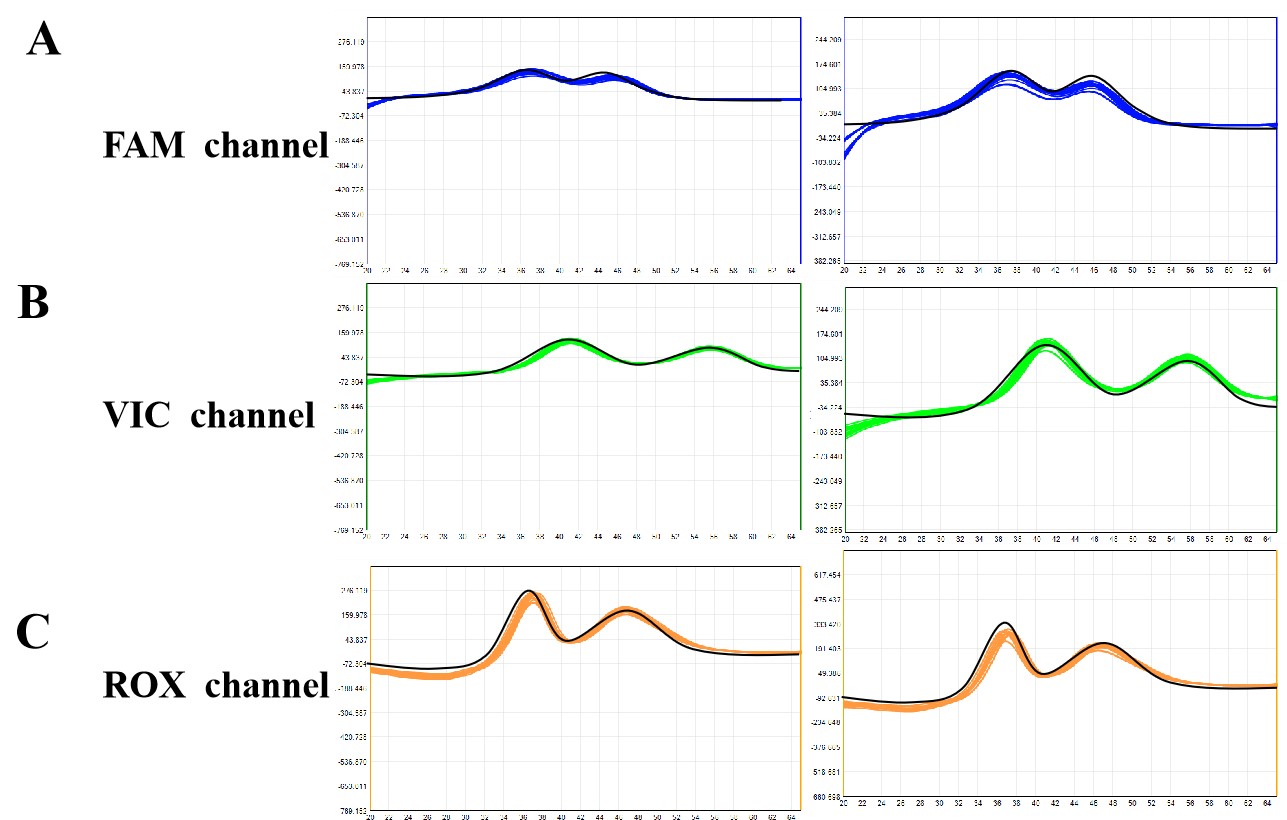


**Figure S3: Repeatability test results of plasmid standards at 100 copies/ml.** (A) FAM channel (blue curve). (B) VIC channel (green curve). (C) ROX channel (orange curve). No specific peaks were detected for all 6 pathogens (0/20 replicates). Abscissa: Temperature; Ordinate: Fluorescence value change rate. Black curves represent negative controls (NCs).


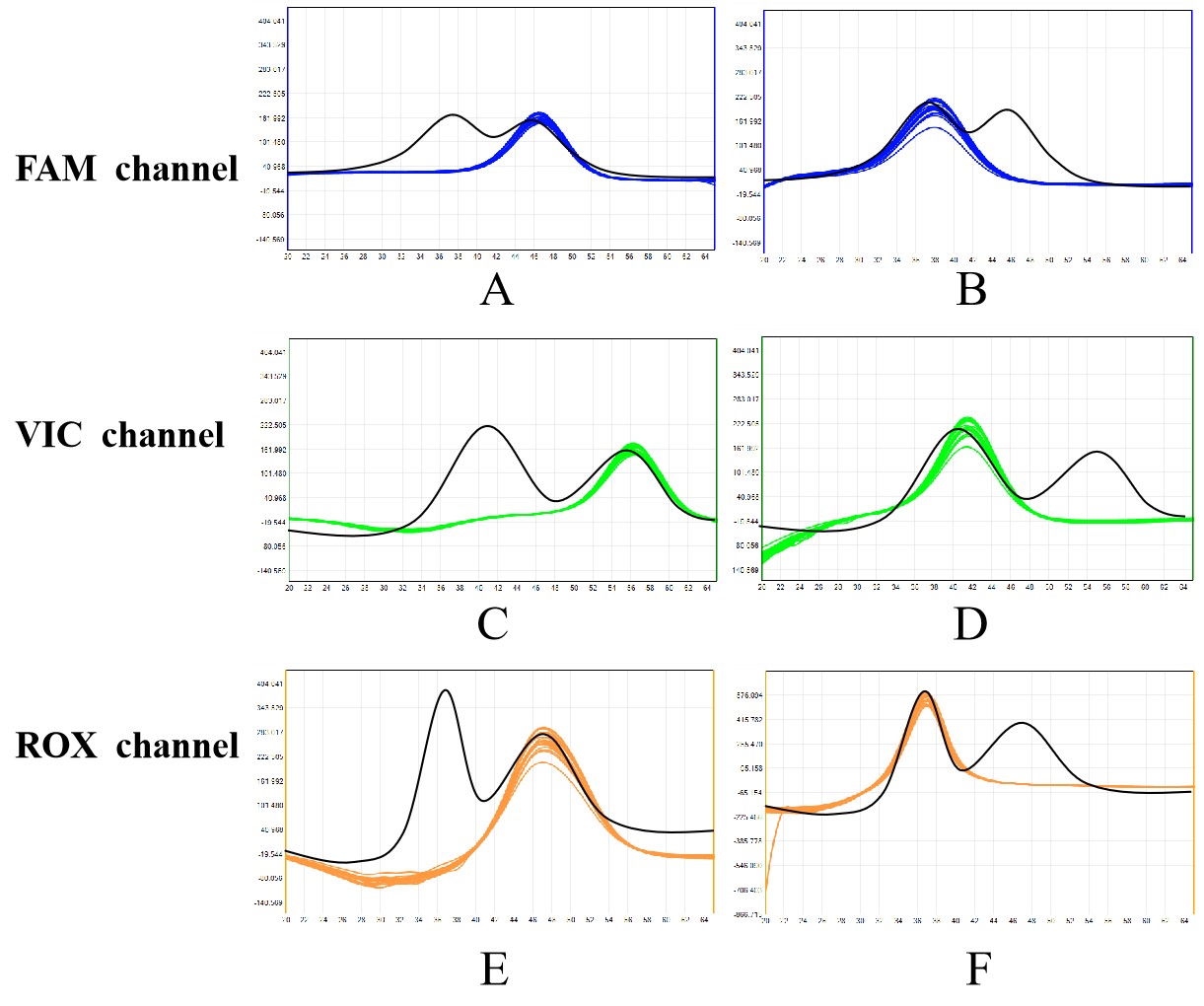


**Figure S4: Melting curve analysis of clinical samples at 200 copies/ml and 500 copies/ml. (**A) HSV-I (200 copies/ml, FAM channel, blue curve). (B) HSV-II (200 copies/ml, FAM channel, blue curve). (C) EBV (500 copies/ml, VIC channel, green curve). (D) HCMV (200 copies/ml, VIC channel, green curve). (E) RV (500 copies/ml, ROX channel, orange curve). (F) TOX (200 copies/ml, ROX channel, orange curve). Abscissa: Temperature; Ordinate: Fluorescence value change rate. Black curves represent negative controls (NCs); blue/green/orange curves represent clinical samples with target peaks indicated by red dashed circles. A/B/D/F correspond to 200 copies/ml (consistent with 100% detection rates in Table 3), and C/E correspond to 500 copies/ml (reference for 100% detection of EBV/RV).


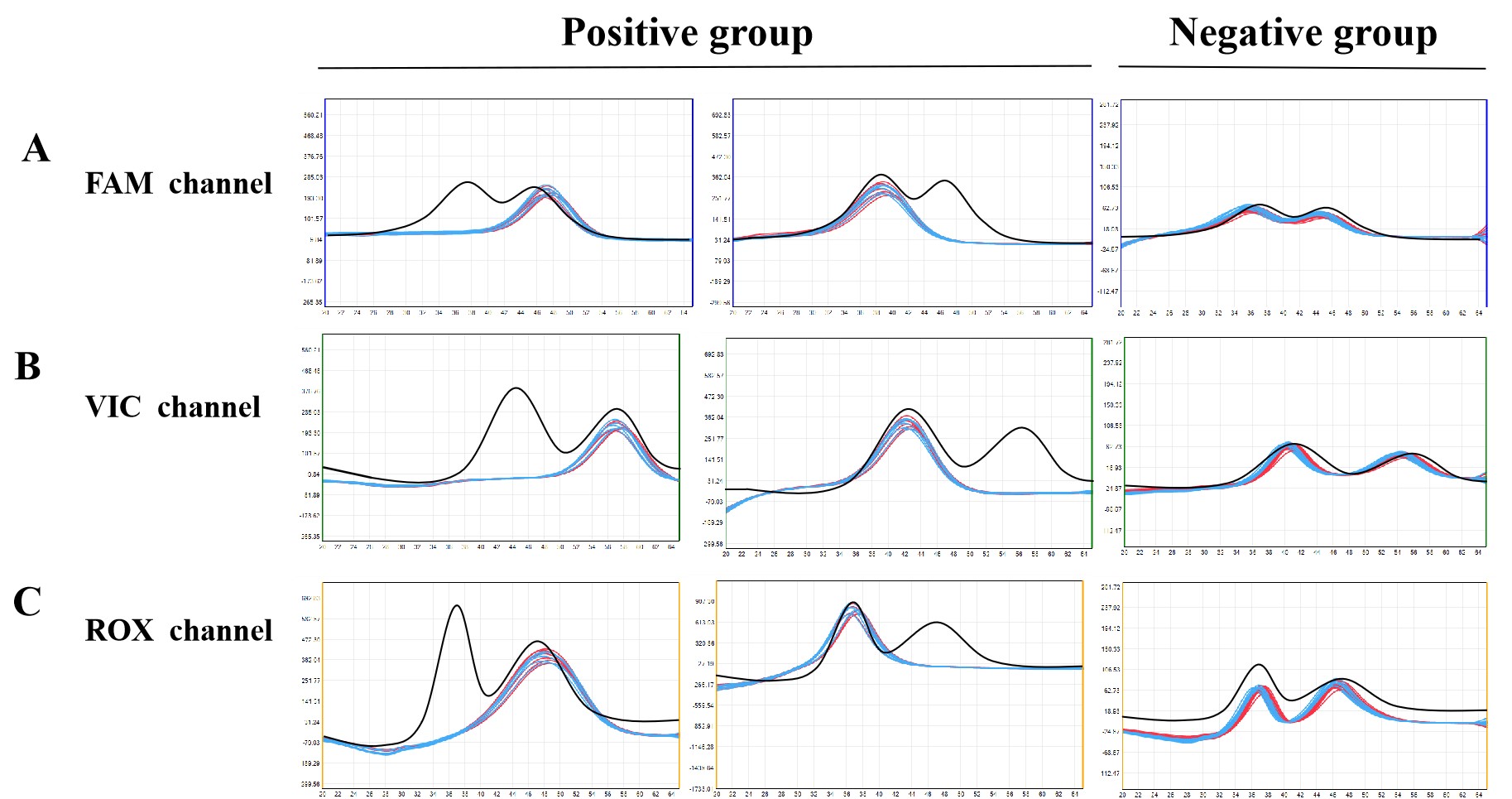


**Figure S5: Effect of interferents on melting curves of TORCH pathogen detection.** (A) FAM channel (HSV-I/HSV-II), Left: Positive group (3×LoD plasmids, including without interferents and with interferents); Right: Negative group (no pathogen, including without interferents and with interferents). (B) VIC channel (EBV/HCMV), Left: Positive group (3×LoD plasmids, including without interferents and with interferents); Right: Negative group (no pathogen, including without interferents and with interferents). ROX channel (RV/TOX), Left: Positive group (3×LoD plasmids, including without interferents and with interferents); Right: Negative group (no pathogen, including without interferents and with interferents).

Abscissa: Temperature; Ordinate: Fluorescence value change rate. No significant Tm shifts (<0.5 ℃) were observed between interferent and non-interferent negative samples (detailed in Table 5), and the target melting peaks disappeared in positive group. In both positive and negative groups, the colored curves represent different tested samples. Black curves represent negative controls (NCs).


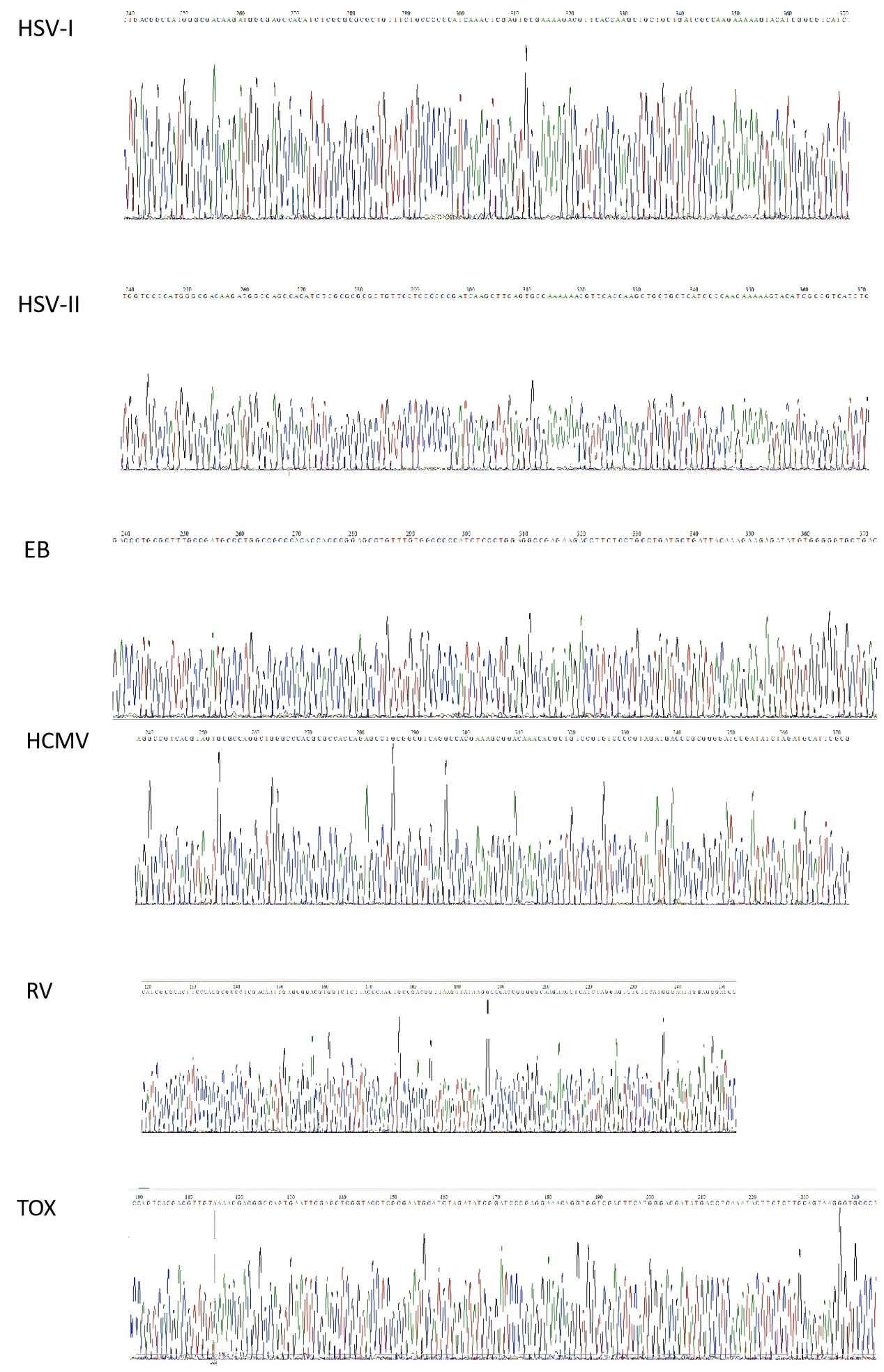


**Figure S6: Sanger Sequencing Validation Results for Discordant Samples.** Seven samples with discordant results between the test method and the control method were validated by Sanger sequencing, confirming the correctness of the test method. The figure shows representative sequencing electropherograms for five pathogen types: HSV-I (1 sample), HSV-II (2 samples), EBV (1 sample), HCMV (1 sample), and RV (2 samples). Colored peaks in the electropherograms correspond to DNA sequence bases (A/T/C/G), with nucleotide positions and sequence information labeled at the top. All validation results were consistent with the pathogen typing results of the test method.
