# Supplementary material for: A streamlined integrated system integrating lysate release, freeze-dried reagents for multiplex polymerase chain reaction, and intelligent analysis for TORCHes pathogen identification
Source: Front Microbiol. 2026 Mar 24;17:1788209. doi: 10.3389/fmicb.2026.1788209 (PMC13053493; doi:10.3389/fmicb.2026.1788209)
Supplement: Supplementary file 2 [file Table_2.DOC]

**Supplementary Table 1 shows the results of testing 50 selected specific samples.**

| Patient ID | HSV-I | HSV-II | EB | CMV | Rv | Tox |
| --- | --- | --- | --- | --- | --- | --- |
| 0001075942 | + |  | + |  | + |  |
| 0000295142 | + |  | + |  | + | + |
| 0001189535 | + |  |  |  |  |  |
| 0001185548 | + |  |  |  |  |  |
| 0000147856 | + |  |  |  |  |  |
| 0001193417 | + |  | + |  |  |  |
| 0000731626 |  | + |  |  |  |  |
| 330799A04896329 |  | + |  | + |  | + |
| 0001071296 |  | + |  | + |  | + |
| 0001191054 |  |  | + |  |  |  |
| 0000617334 |  |  | + |  |  |  |
| 0001197109 |  |  | + |  |  |  |
| 0000418546 |  |  | + |  |  |  |
| 0000866017 |  |  | + |  |  |  |
| 0000585309 |  |  | + |  |  |  |
| 0000417907 |  |  | + |  |  |  |
| 0000595401 |  |  | + |  | + |  |
| 0001212958 |  |  | + |  |  |  |
| 0001183004 |  |  | + |  | + |  |
| 0001005377 |  |  | + |  |  |  |
| 0001215982 |  |  | + |  | + |  |
| 0001216338 |  |  | + |  | + |  |
| 0000943129 |  |  | + |  | + |  |
| 0000193884 |  |  | + |  |  |  |
| 0001139023 |  |  | + |  | + |  |
| 0000619390 |  |  | + |  |  |  |
| 0000890542 |  |  | + |  |  |  |
| 0001143683 |  |  | + |  | + |  |
| 0001203599 |  |  | + |  | + |  |
| 0000740554 |  |  | + |  | + |  |
| 0000611063 |  |  | + |  | + |  |
| 0001130953 |  |  | + |  |  |  |
| 0000650056 |  |  | + |  | + |  |
| 0001132746 |  |  | + |  |  |  |
| 0000637525 |  |  | + |  |  |  |
| 0001105488 |  |  | + |  |  |  |
| 0000624829 |  |  | + |  |  |  |
| 0001122089 |  |  | + |  |  |  |
| 0000784453 |  |  | + |  |  |  |
| 0001133811 |  |  | + |  |  |  |
| 0001133757 |  |  | + |  |  |  |
| 0000314769 |  |  | + |  |  |  |
| 330700GD150457X |  |  |  | + |  |  |
| 0000694037 |  |  |  | + |  |  |
| 0001190703 |  |  |  | + |  |  |
| 0001169587 |  |  |  | + |  |  |
| 0001203563 |  |  |  | + |  |  |
| 0001213870 |  |  |  | + |  |  |
| 32100080057012865570 |  |  |  | + |  |  |
| 330799A20869830 |  |  |  |  |  | + |

**Supplementary Table 2 Performance agreement by clinical sample type.**

| **Sample Type** | **Number of Samples** | **Number of Positive Samples*** | Agreement with Fluorescent PCR | **Kappa Value**  **(95% CI)** |
| --- | --- | --- | --- | --- |
| Whole Blood | 157 | 87 | 154/157 (98.1%) | 0.982 (0.963 - 1.000) |
| Urine | 17 | 9 | 17/17 (100%) | 0.956 (0.870 - 1.000) |
| Herpes Fluid | 13 | 11 | 13/13 (100%) | 0.973 (0.898 - 1.000) |
| Cerebrospinal Fluid (CSF) | 6 | 3 | 6/6 (100%) | 0.967 (0.871 - 1.000) |
| Amniotic Fluid | 5 | 2 | 5/5 (100%) | 0.980 (0.904 - 1.000) |
| Swabs (Throat/Urethral) | 12 | 6 | 12/12 (100%) | 0.971 (0.902 - 1.000) |
| **Total** | **210** | **118** | **207/210 (98.6%)** | **0.988 (0.975 - 1.000)** |

*Positive for one or more of the six TORCH pathogens.
